# Population Pharmacokinetics and Pharmacodynamics of Isoniazid and its Metabolite Acetylisoniazid in Chinese Population

**DOI:** 10.3389/fphar.2022.932686

**Published:** 2022-07-19

**Authors:** Bing Chen, Hao-Qiang Shi, Meihua Rose Feng, Xi-Han Wang, Xiao-Mei Cao, Wei-Min Cai

**Affiliations:** ^1^ Department of Pharmacy, Ruijin Hospital, School of Medicine, Shanghai Jiaotong University, Shanghai, China; ^2^ Department of Pharmaceutical Sciences, University of Michigan, Ann Arbor, MI, United States; ^3^ Department of Clinical Pharmacology, Nanjin Jinling Hospital, Nanjing, China; ^4^ Department of Clinical Pharmacy and Pharmaceutical Management, School of Pharmacy, Fudan University, Shanghai, China

**Keywords:** isoniazid, acetylisoniazid, population pharmacokinetics, N-acetyltransferase 2, genetic polymorphism, pharmacodynamics

## Abstract

**Objective:** We aimed to establish a population pharmacokinetic (PPK) model for isoniazid (INH) and its major metabolite Acetylisoniazid (AcINH) in healthy Chinese participants and tuberculosis patients and assess the role of the *NAT*2 genotype on the transformation of INH to AcINH. We also sought to estimate the INH exposure that would achieve a 90% effective concentration (EC_90_) efficiency for patients with various *NAT2* genotypes.

**Method:** A total of 45 healthy participants and 157 tuberculosis patients were recruited. For healthy subjects, blood samples were collected 0–14 h after administration of 300 mg or 320 mg of the oral dose of INH; for tuberculosis patients who received at least seven days therapy with INH, blood samples were collected two and/or six hours after administration. The plasma concentration of INH and AcINH was determined by the reverse-phase HPLC method. *NAT2* genotypes were determined by allele-specific amplification. The integrated PPK model of INH and AcINH was established through nonlinear mixed-effect modeling (NONMEM). The effect of *NAT2* genotype and other covariates on INH and AcINH disposition was evaluated. Monte Carlo simulation was performed for estimating EC90 of INH in patients with various *NAT*2 genotypes.

**Results:** The estimated absorption rate constant (K_a_), oral clearance (CL/F), and apparent volume of distribution (V_2_/F) for INH were 3.94 ± 0.44 h^−1^, 18.2 ± 2.45 L⋅h^−1^, and 56.8 ± 5.53 L, respectively. The constant of clearance (K_30_) and the volume of distribution (V_3_/F) of AcINH were 0.33 ± 0.11 h^−1^ and 25.7 ± 1.30 L, respectively. The fraction of AcINH formation (F_M_) was 0.81 ± 0.076. *NAT2* genotypes had different effects on the CL/F and F_M_. In subjects with only one copy of NAT2 *5, *6, and *7 alleles, the CL/F values were approximately 46.3%, 54.9%, and 74.8% of *4/*4 subjects, respectively. The F_M_ values were approximately 48.7%, 63.8%, and 86.9% of *4/*4 subjects, respectively. The probability of target attainment of INH EC_90_ in patients with various NAT2 genotypes was different.

**Conclusion:** The integrated parent-metabolite PPK model accurately characterized the disposition of INH and AcINH in the Chinese population sampled, which may be useful in the individualized therapy of INH.

## Introduction

Tuberculosis remains a serious public health concern in many countries (Ferguson and Rhoads, 2009; Sandhu, 2011). Although the incidence of tuberculosis has been decreasing recently, approximately 10 million cases, along with approximately 1.2 million deaths associated with the disease have been reported (Chakaya et al., 2021). China accounted for 8.4% of total global tuberculosis cases in 2019 (Chakaya et al., 2021). Isoniazid (INH), inhibits the synthesis of long-chain mycolic acids, which are indispensable components of mycobacterial cell walls. As such, it is an essential component of standard first-line prophylactic treatment/measures of tuberculosis (Winder and Collins, 1970; Takayama et al., 1972). INH is also effective in preventing resistance to co-administered anti-tuberculosis drugs (Mitchison, 1979). A fixed-dose combination (FDC) anti-tuberculosis medication is thought to be more effective and convenient, with less drug resistence occurring (Blomberg et al., 2001). The use of FDCs from different manufacturers may cause high pharmacokinetic (PK) variability, along with inter-individual differences in efficacy and toxicity of INH (Shishoo et al., 2001; McIlleron et al., 2006; Hao et al., 2014).

Therapeutic drug monitoring (TDM) of anti-tuberculosis drugs, including INH, can be used to ensure adequate treatment effect, as well as reduce toxic effects (Peloquin, 2002; Weiner et al., 2003; Zuur et al., 2016). A peak INH concentration (C_max_) of *<*3 μg ml^−1^ after a daily administration, or *<*9 μg ml^−1^ after biweekly administration is considered ineffective (Peloquin, 2002). Some other studies suggested that a minimum area of 10.52 μg⋅h⋅ml^−1^ under the concentration-time curve (AUC_0–24_) is a suitable marker of clinical efficacy (Donald et al., 2007; Kiser et al., 2012).

The majority of INH is acetylated by *N*-acetyltransferase (NAT2) to form acetylisoniazid (AcINH), acetylhydrazine, and other metabolites, and only approximately 7–30% of INH is excreted intact as the parent drug. Although there is no evidence on the toxicity of AcINH, it may be converted to other toxic metabolites. The ratio of AcINH and INH levels in plasma at the peak time is considered to be an index of INH toxicity (Timbrell et al., 1980). NAT2 activity exhibits remarkable polymorphism, primarily due to *NAT2* genotypes. A total of seven single nucleotide polymorphisms (SNPs) have been determined in the human *NAT2* gene, consisting of more than 27 different *NAT2* alleles (Lauterburg et al., 1985a; Bell et al., 1993; Loktionov et al., 2002; Walker et al., 2009). Two of these SNPs are silent, and the other five lead to amino acid substitutions. Hein et al. found that the C341→T (rs1801280) of *5, G590→A (rs1799930) of *6, and G857→A (rs1799931) of *7 led to substitutions of Ile114→Thr, Arg197→Gln, Gly286→Glu in the NAT2 enzyme, respectively, leading to a remarkable decrease in NAT2 activity (Hein et al., 1994). *NAT2* alleles *5, *6, and *7 comprise the most important factor of slow acetylators in Whites and Asisans. The frequencies of these alleles are different among various populations (Lin et al., 1993; Agundez et al., 1996; Chen et al., 2006). Polymorphism of *NAT2* has been considered as the primary reason for inter-individual differences in PK and pharmacodynamics (PD), which may further influence the INH dosing regimen (Horai et al., 1982; Deguchi et al., 1990; Parkin et al., 1997; Denti et al., 2015).

Population PK (PPK) is suitable for characterizing the disposition of the drug in a large group of participants with limited observations for each. PPK has the ability to provide quantitative estimates of the inter- and intra-patient variability, and also to determine the influence of demographic, clinical, and genetic factors on the PKs. PPK is also used in the simulation of PKs of parent drugs and their metabolites simultaneously (Seng et al., 2015; Sundell et al., 2020). However, to our knowledge, no study has been published on the integrated PPK model of INH and AcINH in Chinese patients. Based on the PPK parameters of INH, and the distribution of minimum inhibitory concentration (MIC) in clinical isolates, the PK/PD model can be established to identify an optimal dosing regimen of INH, which is helpful in the appropriate treatment of tuberculosis (Pasipanodya and Gumbo, 2011).

The purpose of this study is to establish a PPK model for INH and its major metabolite AcINH in tuberculosis patients and healthy Chinese participants,and assess the influence of *NAT2* polymorphism on the PK/PD of INH.

## Materials and Methods

### Subjects

All healthy participants were enrolled in two different PK studies of INH. In the first study, 24 healthy male subjects with different *NAT2* genotypes were recruited from 215 participants with known *NAT2* genotypes (Chen et al., 2009). In the second study, 21 healthy subjects were enrolled from a group that participated in a bioequivalence study. All subjects were Han nationals. Each subject was physically healthy and had no history of significant medical illness or hypersensitivity to any drugs. A physical examination was performed before the study. This included a complete blood chemistry, urinalysis, and an electrocardiogram. All subjects were drug-free for two weeks before, and during the study. The study protocol was approved by the Jinling Hospital Ethics Committee and all subjects gave written consent to participate.

A total of 157 tuberculosis patients were also recruited. The subjects consisted of 89 males and 68 females (age: 42.2 ± 11.5 y, weight: 56.5 ± 9.71 kg) patients. Sixty-seven patients had taken three anti-tuberculosis drugs simultaneously, 48 had consumed four drugs simultaneously, while consumed two drugs simultaneously. The drugs used in combination with INH were rifampicin, rifapentine, pyrazinamide, and ethambutol. Demographic and clinical data were recorded.

### Study Protocol

For the first PK study, 24 healthy volunteers were enrolled in the study center at 8:00 p.m. on the day before the study began. After fasting overnight, three tablets of INH (100 mg/tablet, Qianjin Pharmaceutical Company, Hunan, China) were taken with 200 ml of water at 8:00 a.m. Food was provided four hours after the INH intake. Three ml of blood was drawn into a sterile anticoagulation tube just before the administration of INH, and 0.25, 0.5, 1, 1.5, 2, 3, 4, 6, 8, 10 and 14 h after.

In the second PK study, Rifater, containing INH 80 mg/tablet (Gruppo Lepetit SPA, Italy) was used as the reference formulation, and INH 80 mg/tablet or 80 mg/capsule (LanBen, Nanjing, China) was used as the test formulation. Twenty-one subjects received a single dose of 320 mg test or reference tablets or capsules after an overnight fast. Both formulations were administered with 200 ml water at 8:00 a.m. Standard meals were provided four and ten hours after drug administration. No other food or drinks containing Xanthine (e.g., tea, coffee, cola) were allowed for subjects during their time in the study center. A total of three ml of blood was collected via veins, and then transferred into sterile EDTA anticoagulation tube before dosing, and 0.25, 0.5, 1, 1.5, 1, 2, 4, 6, 9, 12 h after.

All patients received anti-tuberculosis therapy for 7–14 consecutive days before sample collection. For all patients, 3 ml of blood was drawn 2 and/or 6 h after INH administration. Blood samples were centrifuged at 1610 × *g* for 10 min. Since AcINH is not stable in plasma samples, plasma was immediately separated and stored at −70°C while awaiting further analysis.

### Genotyping

*NAT2* C341→T, G590→A, and G857→A represent *5, *6, and *7 alleles, respectively. These SNPs are sufficient for the reflection of the important *NAT2* genotypes have impaction on NAT2 activity. We developed allele-specific PCR (ASA-PCR) to determine the three aforementioned SNPs (Chen et al., 2004). The method was designed based on the principles of amplification refractory mutation systems. Briefly stated, allele-specific primers for different *NAT2* alleles were designed. ASA-PCR was performed using two tubes for the detection of the wild and mutant genotype of each allele. The primers were mixed with Taq DNA enzyme, dNTPs, Mg^2+^, buffer, and genomic DNA. Upon 30 amplification cycles, the *NAT2* genotypes were analyzed after electrophoresis.

### Quantification of INH and AcINH

A previously established reverse phase HPLC was used to determine plasma concentrations of INH and AcINH simultaneously (Cao et al., 2005). Perchloric acid was added to the plasma for precipitation of protein. The supernatant was separated after centrifugation and eluted with 2 mmol⋅L^−1^ sodium heptane sulfonate-acetonitrile (98:2) on a Lichrospher C18 column and detected at a wavelength of 266 nm. The calibration curve was linear in the range of 0.12–15.89 mg⋅L^−1^ (0.88–115.9 μmol⋅L^−1^) for INH and in the range of 0.13–17.08 mg⋅L^−1^ (0.72–95.4 μmol⋅L^−1^) for AcINH (r^2^ > 0.99).

### Non-Compartmental Analysis

The PK parameters of INH and AcINH in healthy subjects were determined with the WinNonlin 5.01 (Pharsight Corp., Mountain View, CA, United States). C_max_ and T_max_ were obtained from observation. The K_e_ was calculated using least-square regression analysis performed using the terminal phase of the concentration–time curve. The AUC was calculated by using the trapezoidal method.

### Compartmental Pharmacokinetic Analysis

#### Data Splitting

For the 202 subjects in the present study, 122 were assigned to the model-building set (index group) and 80 to the test set (validation group), respectively. The index group included 15 subjects with one full PK profile, 12 subjects with three full PK profiles, and 95 patients with sparse sampling data. The validation group includednine subjects with one full PK profile, nine with three full PK profiles, and 62 with sparse sampling data.

### Model Development

PPK modeling of INH and AcINH was constructed based on the of modeling group data. The final PPK model was constructed by using data from both the modeling group and validation group. One- and two-compartment models for INH or AcINH were evaluated during model construction. The first absorption, with or without lag time, was also tested. NONMEM (Version 6, GloboMax, Hanover, MD) was used for the model construction. The first-order conditional estimation method (FOCE) was applied. Model selection was based on objective function value (OFV), parameter estimates, and standard error. OFV is proportional to −2 log likelihood of the relevant model. The distribution of empirical Bayes estimates and ability of the model to determine reasonable INH and AcINH concentration-time profiles were also important for model assessment.

### Interindividual and Residual Error Model

The inter-individual variability of the parameters was assessed using an exponential function (Eq. 1)

$${\text{P}}_{\text{i}}=\theta_{\text{TV}}\cdot {\text{e}}^{\eta \text{i}}$$ (1)

*P*_*i*_ denotes the PK parameter value, *θ*_*TV*_ denotes the population value for the parameter described, and η_i_ denotes the random deviation of *P*_*i*_ from *θ*_*TV*_. The values of η_i_ were assumed to be independently normally distributed with a mean of 0 and a variance of ω^2^.

A proportional model was used for residual error analysis of INH and AcINH as (Eq. 2)

$${\text{C}}_{\text{obs}}{\;=\text{ C}}_{\text{pred}}\text{×}\left(1+\;\varepsilon \right)$$ (2)

C_obs_ is the observed concentration, C_pred_ is the predicted concentration, and ε is a residual error with a mean of 0 and a variance of σ^2^.

### Covariates

The characteristics of the subjects, including body weight, age, clearance of creatinine (CLcr), aspartate aminotransferase (ASP), alanine aminotransferase (AST), and *NAT2* genotype, were tested as covariates in the population analysis. The model of continuous covariates on the *P*_*i*_ was as follows (Eqs 3–6)

$$\text{TV}\left({\text{P}}_{\text{i}}\right)=\theta_{\text{P}}\text{×}\left(\text{covariate}\right)$$ (3)

$$\text{TV}\left({\text{P}}_{\text{i}}\right)=\theta_{\text{P}}+\theta_{\text{C}}\text{×}\left(\text{covariate}\right)$$ (4)

$$\text{TV}\left({\text{P}}_{\text{i}}\right)=\theta_{\text{P}}{\text{×e}}^{\left(\text{covariate×}\theta_{\text{c}}\right)}$$ (5)

$$\text{TV}\left({\text{P}}_{\text{i}}\right)=\theta_{\text{P}}\text{×}{\left(\text{covariate}/\text{means of covariate}\right)}^{\theta \text{c}}$$ (6)

For categorical covariate of the *NAT2* genotype, three methods representing *NAT2* genotyping were tested:

1) Subjects were placed into extensive metabolizer (EM) and poor metabolizer (PM) groups according to the NAT2 genotype, where scores of “1” and “0” were assigned to the EM and PM subjects, respectively.
2) Subjects were characterized as homogenous wild type (wt/wt), heterozygous mutant (m/wt), and homogenous mutant (m/m) based on their genotypes. Scores of “0,” “1,” and “2” were assigned to the three groups, respectively. *P*_*i*_ was modeled according to the following equation (Eq. 7)

$${\text{P}}_{\text{i}}=\text{TV}\left({\text{P}}_{\text{i}}\right){\text{×e}}^{\left(\text{NAT}2\text{×}\theta \right)}$$ (7)

3) For each allele of *5, *6 and *7, scores of 0, 1 and 2 were assigned to the w/w, m/w, and m/m genotype. Hence, subjects with *4/*4, *4/*6, *4/*7, *6/*6, *6/*7, *7/*7, and *5/*7 genotype were assigned the scores of 0/0/0, 0/1/0, 0/0/1, 0/2/0, 0/1/1, 0/0/2, and 1/0/1, respectively. *P*_*i*_ was modeled according to the following equation (Eq. 8)

$${\text{P}}_{\text{i}}=\text{TV}\left({\text{P}}_{\text{i}}\right){\text{×e}}^{\left(\text{*}5\text{×}\theta \right)}{\text{×e}}^{\left(\text{*}6\text{×}\theta \right)}{\text{×e}}^{\left(\text{*}7\text{×}\theta \right)}$$ (8)

Various covariates were included in a stepwise manner, followed by a stepwise elimination for the final regression model. Changes in the OFV were used to approximate the χ^2^ distribution, with degrees of freedom (df) equal to the number of covariates introduced. When the OFV was reduced by 6.64 or higher (*p* < 0.01, df = 1), the covariate included was considered statistically significant. After all covariates significantly decreasing OFV were included, each covariate was fixed as zero in turn. This procedure was repeated until the value of the objective function failed to increase by more than 10.9 (*p* < 0.001, df = 1).

### Data Analysis and Model Evaluation

The final model was tested by using a visual predictive check. A total of 1,000 new datasets for patients with different *NAT2* genotypes were simulated based on estimation of the parameters with their inter- and intra-individual variety, and *NAT2* genotypes of healthy subjects and tuberculosis patients. A 90% prediction interval was obtained by extracting the 5% and 95% quantiles of their simulated distributions. The quality of the evaluation was assessed by graphical presentation of the observed concentration *vs.* predictions. The stability and performance of the final model were also assessed through a nonparametric bootstrap, with resampling and replacement. In this study, 1,000 bootstrap samples were generated, and the PPK parameters were estimated for each sample using the final model.

### Bayesian Estimation of AUC of INH

Based on the Bayesian approach, the POSTHOC subroutine of NONMEM without an estimation step (MAXEVAL = 0) was used to estimate the individual PK parameters of each patient in the validation group. The PPK parameters, inter-individual variation (IIV), and residual variability were set as the data obtained from the index group. Models of INH C_2_, C_2_-C_4_, and C_2_-C_6_ plasma concentration-time points following INH administration were selected as the Bayesian estimators for estimation of the AUC of the validation group. AUC_0-24_ was estimated through the equation: AUC_0-24_ = dose/(CL/F). AUC_0-24_ obtained from the Bayesian estimators was compared with observed values. The predictive performance was evaluated as bias and precision, which are expressed as MPE (%) and RMSE (%), respectively.

### Monte Carlo Simulations

In line with previous studies, AUC_0–24_/MIC indicated a better PK/PD index compared with C_max_/MIC and T_MIC_. The 90% maximal kill *Mycobacterium tuberculosis* (90% effective concentration, EC_90_) is considered the therapeutic target of INH. AUC_0–24_/MIC that mediates EC_90_ was 567 (Gumbo et al., 2007). The MIC of INH was obtained from previous studies on *Mycobacterium tuberculosis* strains (MIC values were 0.01, 0.02, 0.05, 0.10, and 0.25 μg/ml) (Zhao and Lu, 2019). The INH dosing regimen included 300, 600, and 900 mg daily. AUC_0–24_ of INH was estimated based on the integrated PPK model with estimated uncertainty (standard error). The simulation was performed with the data of 10,000 subjects with various *NAT2* genotypes using the Monte Carlo method (Gumbo. 2010). The probability of target attainment (PTA) of patients with various *NAT2* genotypes was summarized based on the estimated proportions of isolates.

## Results

### INH and AcINH in Relation to *NAT2* Genotypes

The demographic and laboratory test data of all subjects obtained are shown in Table 1. In the first PK study performed with 24 healthy subjects, five different *NAT2* genotypes were observed: *4/*4 (8), *4/*6 (6), *4/*7 (2), *5/*7 (1), and *6/*6 (7). A total of eight subjects were characterized with a wt/wt genotype (*4/*4);eight with m/wt genotype (*4/*6 and *4/*7); 8 subjects with m/m genotype (*5/*7 and *6/*6). There was a significant difference in INH PK parameters among various groups. The C_max_ of INH in the wt/wt, m/wt, and m/m groups were 36.0 ± 13.5, 41.7 ± 14.2, and 60.4 ± 17.5 μmol⋅L^−1^, and AUC_0–14h_ of INH in different groups were 71.5 ± 16.8, 115.6 ± 21.9, and 274.9 ± 55.5 μmol⋅h⋅L^−1^ (*p* < 0.001), respectively (Table 2). A significant difference was observed among various *NAT2* genotypes.

**TABLE 1:** Demographic data of pharmacokinetic study on healthy subjects and tuberculosis patients.

Characters	Mean ± SD		
	PK study	Bioequivalence study	Patients
Gender	Male: 21; Famale: 0	Male: 24; Famale: 0	Male: 89; Famale: 68
Age (year)	24.1 ± 2.61 (21–29)	24.4 ± 2.12 (22–28)	42.2 ± 11.5 (19–64)
weight (kg)	63.0 ± 5.82 (55–72)	64.6 ± 5.82 (55–71)	56.5 ± 9.7 (39–78) kg
Cretinine (mmol×L^−1^)	98.7 ± 11.7 (80–124)	69.1 ± 15.2 (52–97)	103.7 ± 25.6 (60–189)
BUN (mmol×L^−1^)	4.40 ± 1.12 (2.9–7.0)	5.21 ± 1.13 (2.8–6.7)	6.8 ± 2.9 (4.1–10.7)
TBIL (mmol×L^−1^)	18.0 ± 7.12 (9.5–32.1)	16.8 ± 3.77 (12.6, 23.3)	11.1 ± 2.11 (6.6–25.5)
ALT (U×L^−1^)	16.3 ± 4.81 (13–28)	14.9 ± 3.63 (11–27)	18.2 ± 8.61 (7–44)
AST (U×L^−1^)	16.8 ± 3.12 (14–28)	18.7 ± 2.31 (17–24)	22.4 ± 7.63 (12–43)

BUN, blood urea nitrogen; TBIL, total bilirubin; ALT, alanine aminotransferase; AST, glutamic-oxaloacetic transaminase.

**TABLE 2:** Pharmacokinetic parameters of INH and AcINH in Chinese healthy subjects with different NAT2 genotypes.

	wt/wt	m/wt	m/m	Total
INH				
t_1/2_ (h)	1.15 ± 0.18	1.76 ± 0.17‡	3.23 ± 0.28‡#	2.05 ± 0.91
C_max_ (μmol ⋅ L^−1^)	36.0 ± 13.5	41.7 ± 14.2	60.4 ± 17.5‡*	46.0 ± 18.0
AUC (μmol ⋅h⋅L^−1^)	75.5 ± 15.5	119.2 ± 22.2†	308.1 ± 62.1‡#	162.1 ± 102.3
Cl (L⋅h^−1^)	30.05 ± 6.02	19.17 ± 5.04	7.79 ± 1.62‡#	19.00 ± 10.28
AcINH				
t_1/2_ (h)	3.84 ± 0.46	3.59 ± 0.40	6.04 ± 1.20‡#	4.57 ± 1.35
C_max_ (μmol ⋅ L^−1^)	32.5 ± 8.03	23.2 ± 2.91†	8.03 ± 1.40 ‡#	21.2 ± 11.3
AUC (μmol ⋅h⋅L^−1^)	245.4 ± 51.2	221.4 ± 31.0	115.1 ± 21.6‡#	192.7 ± 69.0

†*p* < 0.05, ‡*p* < 0.001 compared with wt/wt genotype. *p < 0.05, #*p* < 0.001 compared with m/wt genotype.

For the bioequivalence study performed with 21 healthy subjects who received 320 mg of INH, five different *NAT2* genotypes were observed, namely, *4/*4 (11), *4/*6 (6), *4/*7 (2), *4/*5 (1), and *6/*6 (1). A total of 11, 9, and 1 subjects were included in the *NAT2* wt/wt, m/wt, and m/m groups, respectively. The INH C_max_ in the wt/wt, m/wt, and m/m groups were 32.4 ± 13.7, 42.6 ± 11.0, and 71.6 μmol⋅L^−1^, and AUC_0–24h_ of INH in different groups were 55.2 ± 10.1, 97.7 ± 16.7, and 319.9 μmol⋅h⋅L^−1^ (*p* < 0.001), respectively.

For 157 tuberculosis patients, seven different *NAT2* genotypes including *4/*4 (*n* = 72), *4/*5 (*n* = 6), *4/*6 (*n* = 31), *4/*7 (*n* = 25), *6/*6 (*n* = 12) and *7/*7 (*n* = 4), *5/*7 (*n* = 2), and *6/*7 (*n* = 5) were observed. A total of 72 (45.8%) patients with *4/*4 (wt/wt) genotype and 23 patients (14.6%) with *5/*7, *6/*7, *6/*6 or *7/*7 genotypes were classified in the m/m group. The other 62 patients (39.5%) were classified in the m/wt group. The concentrations of INH 2 h (*n* = 109) after the administration in the wt/wt, m/wt, and m/m groups were 22.8 ± 17.3, 28.2 ± 23.2, and 46.8 ± 28.8 μmol⋅L^−1^ (*p* < 0.001), and the concentrations of AcINH in the three groups were 35.0 ± 18.8, 21.5 ± 15.8, and 11.9 ± 8.64 μmol⋅L^−1^ (*p* < 0.001), respectively. The concentrations of INH 6 h (*n* = 52) after administration in the wt/wt, m/wt, and m/m groups were 3.96 ± 1.74, 6.69 ± 3.56, and 24.2 ± 9.95 μmol⋅L^−1^ (*p* < 0.001), and the concentrations of AcINH in the three groups were 21.3 ± 13.5, 18.1 ± 15.2, and 9.87 ± 3.35 μmol⋅L^−1^ (*p* = 0.034), respectively.

### Integrated Model of INH and AcINH

**FIGURE 1:**
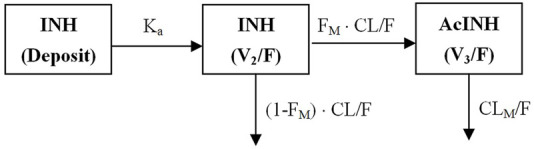
Schematic figure of the integrated PPK model of isoniazid (INH) and acetylisoniazid (AcINH). K_a_: The absorption rate constant of INH. F: The oral bioavailability of INH. CL/F: clearance of INH. F_M_: The fraction of INH converted to AcINH. CL_M_/F: clearance of AcINH. V_2_ and V_3_: volume of distribution of INH and AcINH.

An integrated model describing INH and AcINH simultaneously was developed. The graphical representation of the model is provided in Figure 1. The structural model consisted of a one-compartment model obtained by first-order elimination process for both INH and AcINH. The PPK parameters of INH and AcINH are presented in Table 3. For the basic model, the K_a_ of INH was estimated to be 3.94 ± 0.44 h^−1^. The clearance of INH (CL/F) was estimated to be 18.2 ± 2.45 L⋅h^−1^, K_30_ (constant of clearance of AcINH) was 0.33 ± 0.11 h^−1^, V_d_/F values of INH (V_2_) and AcINH (V_3_) were 56.8 ± 5.53 L and 25.7 ± 1.30 L, respectively, and the F_M_ was 0.81 ± 0.076.

**TABLE 3:** Parameters of integrated PPK model of INH and AcINH in Chinese healthy subjects and tuberculosis patients.

		Basic Model		Final Model (1)		Final Model (2)		Bootstrap	
		Base	SE	Final	SE	Final	SE	mean	SE
K_a_ (1/h)	θ_1_	3.94	0.44	3.91	0.44	3.99	0.42	3.96	0.45
CL/F (L/h)	θ_2_	18.2	2.45	28.7	3.22	30.2	3.04	32.2	3.55
K_30_ (1/h)	θ_3_	0.33	0.11	0.41	0.051	0.30	0.024	0.29	0.033
V_2_/F (L)	θ_4_	56.8	5.53	54.1	12.5	51.4	3.28	56.9	5.14
V_3_/F (L)	θ_5_	25.7	1.30	17.2	3.21	12.4	0.41	13.0	3.13
F_M_	θ_6_	0.73	0.076	0.88	0.21	0.86	0.34	0.86	0.31
M341 (CL)	θ_7_			−0.55	0.11	−0.77	0.119	−0.77	0.22
M590 (CL)	θ_8_					−0.60	0.066	−0.61	0.067
M870 (CL)	θ_9_					−0.29	0.095	−0.27	0.011
M341 (F_M_)	θ_10_			−0.47	0.17	−0.72	0.040	−0.70	0.042
M590 (F_M_)	θ_11_					−0.45	0.046	−0.47	0.049
M870 (F_M_)	θ_12_					−0.14	0.069	−0.14	0.074
IIV									
ωK_a_ (%)	η_1_	51.4	14.3	55.2	43.7	53.5	13.1	47.8	11.4
ωCL/F (%)	η_2_	56.6	21.8	30.7	12.9	28.1	11.1	25.9	10.2
ωK_30_ (%)	η_3_	20.4	9.85	21.4	12.0	22.1	8.15	16.3	8.57
ωV_2_/F (%)	η_4_	21.3	7.32	19.4	4.85	19.8	8.55	20.2	7.72
ωF_M_ (%)	η_5_	35.3	15.1	10.7	5.52	9.82	4.74	6.35	4.73
Residual variance	σ_INH_	33.9	11.0	33.3	13.4	33.9	11.4	31.9	12.4
	σ_AcINH_	29.5	11.9	30.2	11.3	29.5	13.7	27.5	11.8

Final Model (1): NAT2 covariate: NAT2 was classified as wt/wt, m/wt, m/m; CL/F = 28.7×e^(−0.55×Genotype)^; F_M_ = 0.88×e^(−0.55×Genotype)^

Final Model (2): NAT2 covariate: NAT2 was classified according to various NAT2 alleles. CL/F = 30.2×e^(−0.77×M341-0.60×M590-0.29×M803)^; F_M_ = 0.86×e^(−0.72×M341-0.45×M590-0.14×M803)^

After the forward inclusion and backward elimination step, only the effect of *NAT2* genotypes remained in the model. When the subjects were characterized with the genotypes of wt/wt, m/wt, or m/m or as EM and PM, the ΔOFV values were -275.3 and -261.4, respectively. When *5, *6 and *7 alleles were used as covariates separately, the OFV value demonstrated the most significant reduction (ΔOFV was −315.5). In subjects with only one copy of *NAT2* *5, *6, and *7 alleles, the CL/F values were approximately 46.3%, 54.9%, and 74.8% of *4/*4 subjects, and F_M_ values were approximately 48.7%, 63.8%, and 86.9% of *4/*4 subjects. After inclusion of the *NAT2* genotype as a covariate, IIV of CL/F and F_M_ values were reduced from 56.6% to 28.1%, and 35.3% to 9.82%, respectively.

**FIGURE 2:**
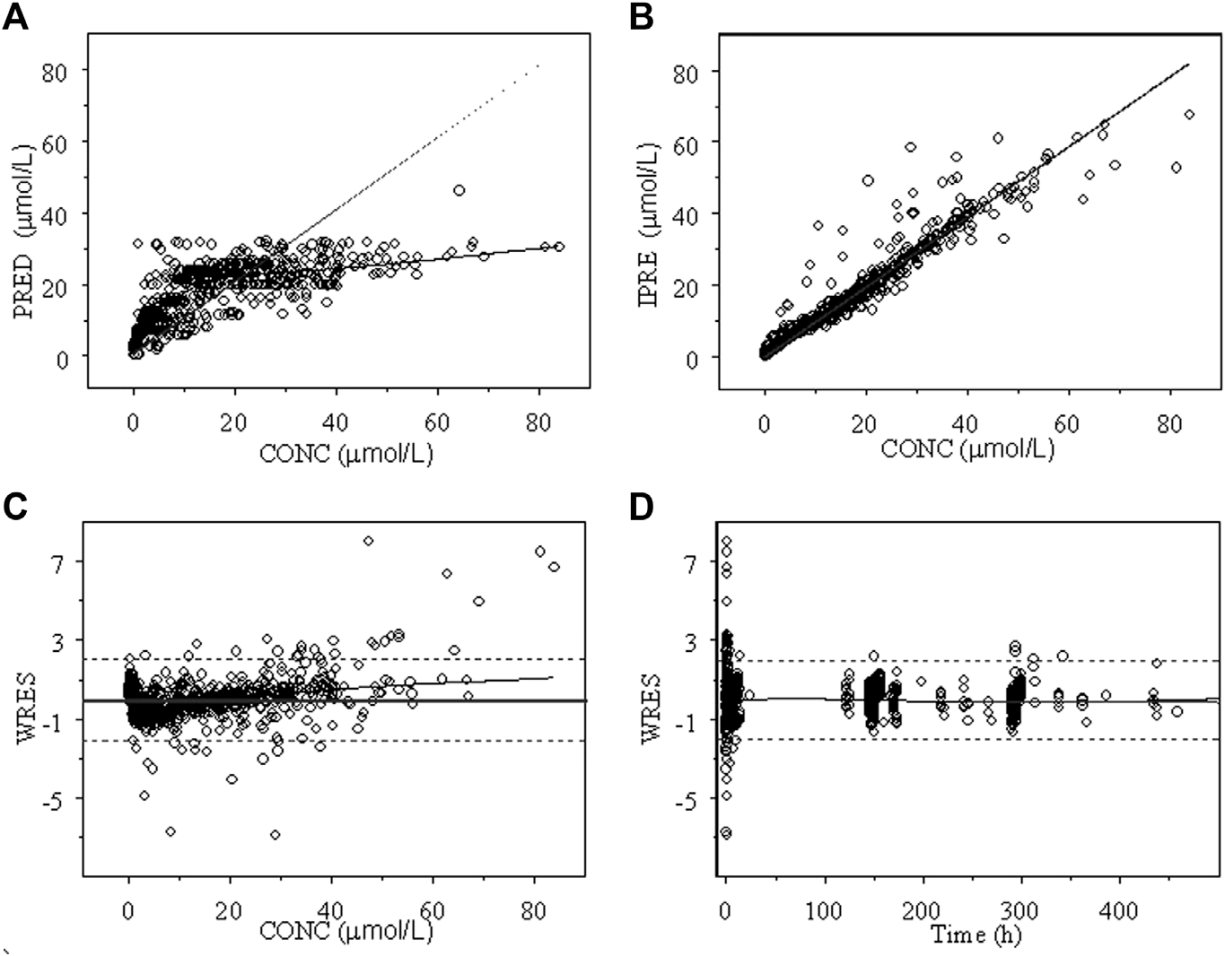
Goodness of fit of INH from the final integrated PPK model in Chinese healthy subjects and tuberculosis patients. **(A)** Population predicted concentration (PRED) vs. observed concentration (CONC); **(B)** individual predicted concentration (IPRE) vs. DV; **(C)** weighted residual error (WRES) vs. CONC; **(D)** WRES vs. time.

**FIGURE 3:**
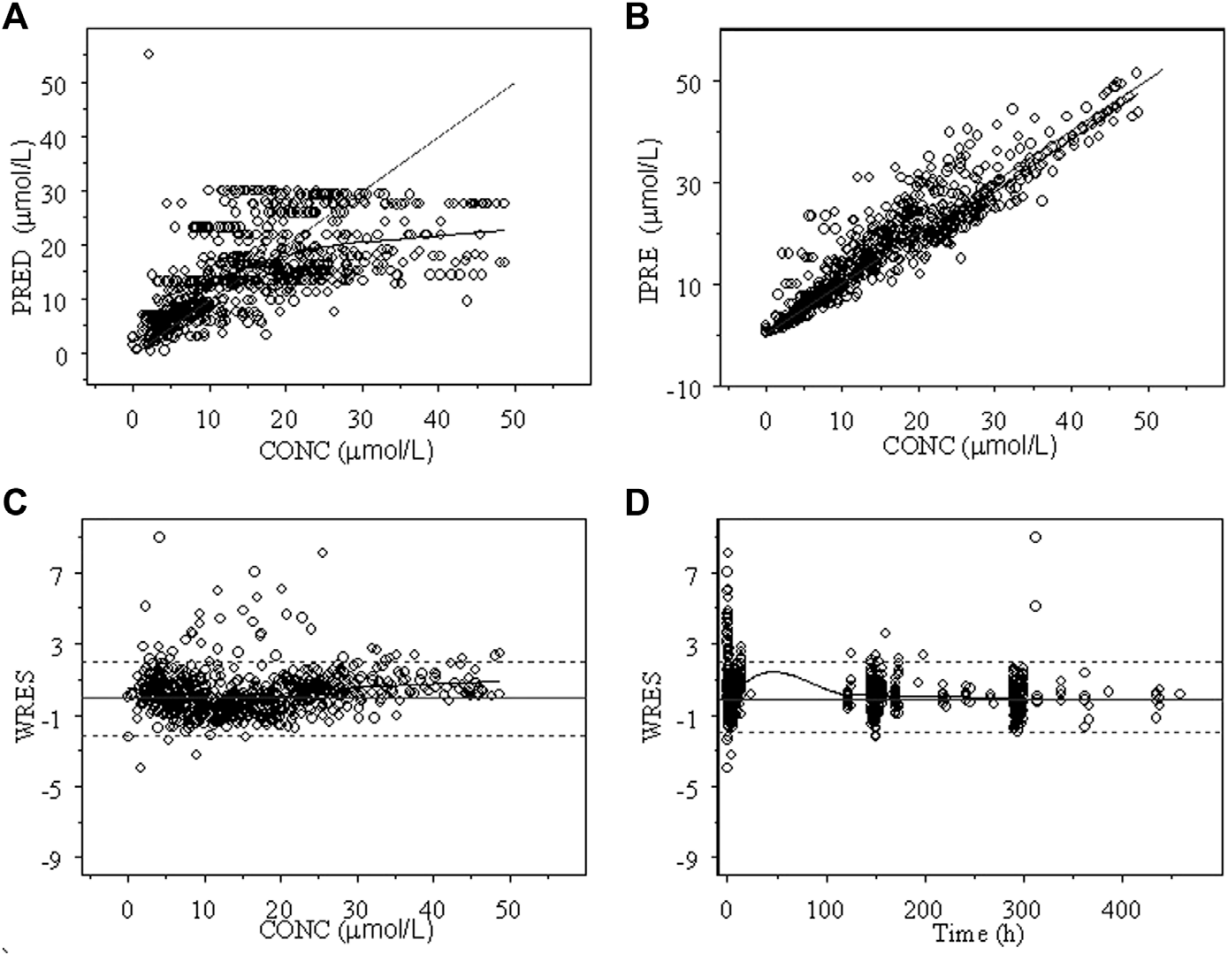
Goodness of fit of AcINH from the final integrated PPK model in Chinese healthy subjects and tuberculosis patients. **(A)** Population predicted concentration (PRED) vs. observed concentration (CONC); **(B)** individual predicted concentration (IPRE) vs. DV; **(C)** weighted residual error (WRES) vs. CONC; **(D)** WRES vs. time.

The goodness of fit plots of the population predicted (PRED) and individual predicted (IPRE) *vs.* observed concentrations (CONC) of the integrated model and the individual weighted residuals (WRES) *vs.* PRED and WRES *vs.* time for INH and AcINH are presented in Figures 2, 3, respectively. The higher bias of INH is distributed in the first two hours. Hence, it seems that the absorption phase was not effectively estimated. The residual errors for INH and AcINH were 33.9% and 29.5%, respectively.

**FIGURE 4:**
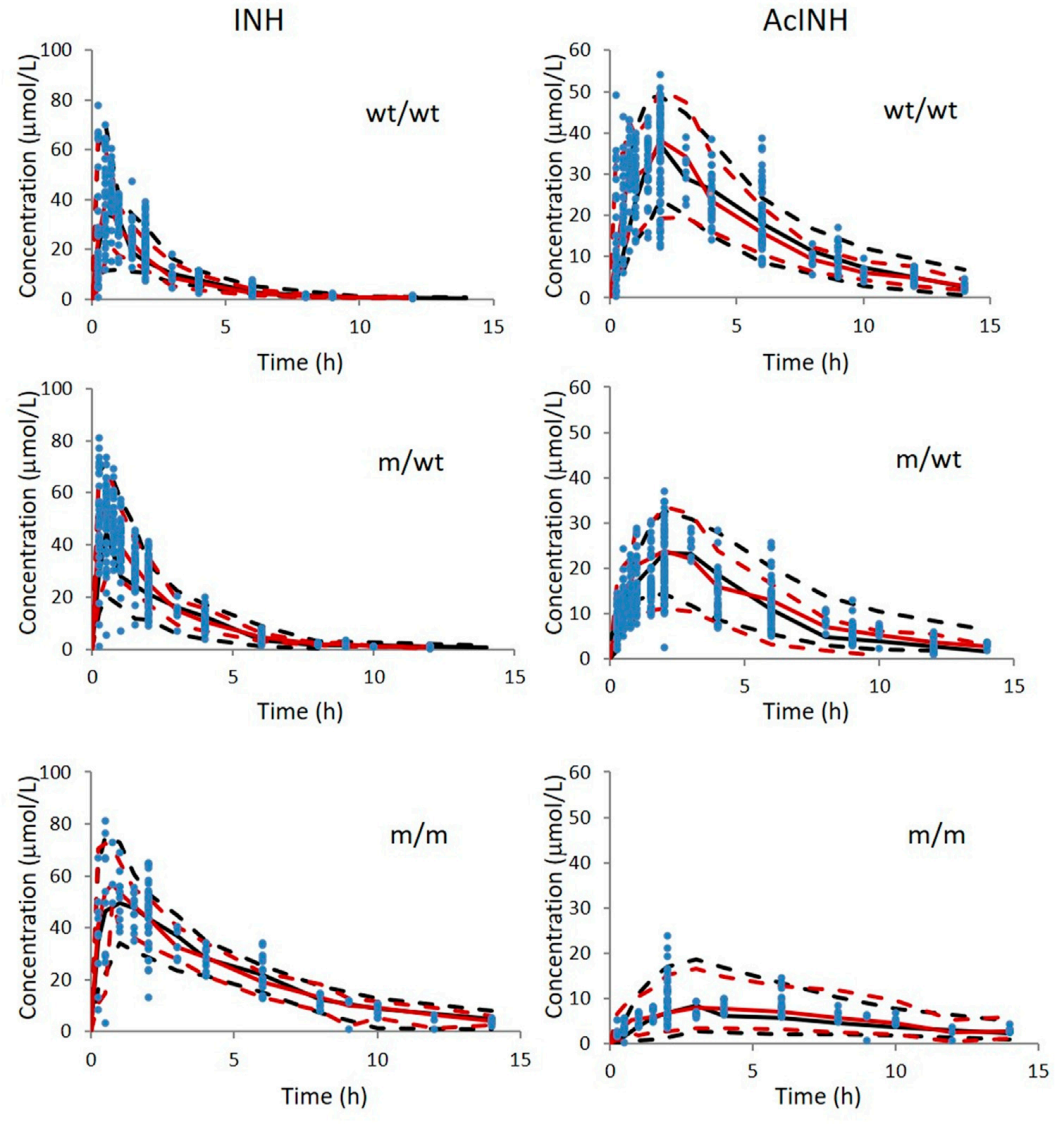
Visual predictive check of INH and AcINH based on the integrated PPK model in Chinese healthy subjects and tuberculosis patients with therapy of INH. The red and black sold lines represent 50th of observed data and simulated data; The upper and lower red dashed lines represent 95th and 5th of observed data; The upper and lower black dashed lines represent 95th and 5th of the simulated data. The blue solid cycles represent observed data wt/wt: NAT2 homogenous wild type; m/wt: NAT2 heterozygous mutant genotype; m/m: homogenous mutant genotype.

VPC from the data set simulated using the final modelisshown in Figure 4. The predicted medians and 5th and 95th data percentiles were similar to the observed medians and 90th percentiles for INH and AcINH. Bootstrap runs were performed successfully 89.7 percent (897 of 1000) of the time similar to the 95% CI calculated by NONMEM.

### Bayesian Estimator

Using Bayesian estimation, the individually predicted INH and AcINH concentrations of the validation group (80 patients, 302 points) by correlated well with observed data. The MPE (95% CI) and MRSE % (95% CI) values were −1.16% (−4.35%, 6.68%) and 27.7% (22.2%, 33.2%) for INH and 2.46% (−0.39%, 5.31%) and 28.6% (23.0%, 34.3%) for AcINH, respectively.

Based on Bayesian method, CL/F of INH obtained from the index group was used to predict AUC_0–24_ in the validation group. The sampling strategies consisted of observation of plasma levels at 2 h (C_2_), 2 and 4 h (C_2_-C_4_), 2 and 6 h (C_2_-C_6_) after INH administration (Table 4). The AUC_0–24_ values of various strategies were 143.4 ± 85.6 μmol⋅h/L (19.7 ± 11.7 μg⋅h/ml) and 122.0 ± 88.1 μmol⋅h/L (16.7 ± 12.1 μg⋅h/ml).

**TABLE 4:** Prediction performance of the selected limited sampling strategy of INH AUC_0–24h_.

	Time Points	r^2^	AUC(μg⋅h/mL)	PE (%)		MRSE (%)	
			Mean ± SD	Mean ± SD	95% CI	Mean ± SD	95% CI
1	2 h	0.825	19.8 ± 9.30	−10.4 ± 18.6	−15.9– −4.90	18.5 ± 24.8	13.9–28.5
2	2–4 h	0.835	21.6 ± 9.89	−2.46 ± 18.6	−7.96–3.04	11.7 ± 28.2	10.26–26.9
3	2–6 h	0.840	21.0 ± 9.93	−5.46 ± 18.2	−10.8– −0.080	12.4 ± 27.2	10.8–26.8

### Monte Carlo Simulation for Patients With Various *NAT2* Genotypes

**FIGURE 5:**
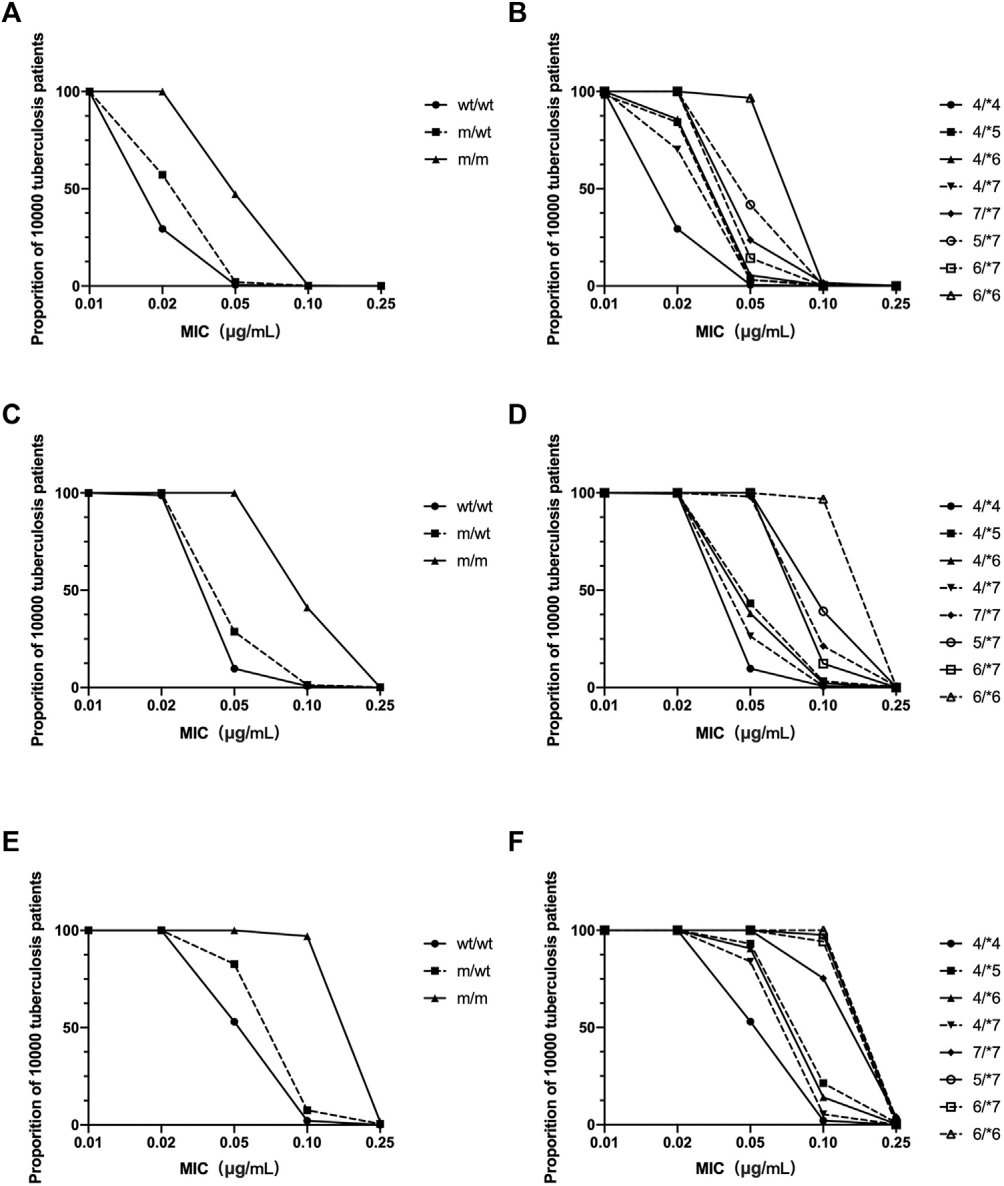
The estimated probabilities of target attainment in patients with various NAT2 genotype and various doing regimens by a Monte Carlo simulation method. INH dosing regimen: 300 mg daily **(A,B)**; 600 mg daily **(C,D)**; 900 mg daily **(E,F)**. **(A,C,E)** NAT2 genotype was characterized as homogenous wild type (wt/wt), heterozygous mutant (m/wt), and homogenous mutant (m/m). **(B,D,F)** NAT2 genotype was characterized according to various *NAT2* alleles.

Using the Monte Carlo simulation method, PTAs for patients with various *NAT2* genotypes were estimated with various INH regimens. It seemed that with a daily regimen including 300 mg of INH, the PTA target could be obtained in patients with wt/wt and m/wt genotypes, with the MIC lower than 0.01 μg/ml. With a daily regimen involving administration of 600 mg INH, target PTA can be obtained with MIC values lower than 0.02 μg/ml in wt/wt and m/wt patients. For m/m patients, target PTA was obtained with MIC values of 0.02, 0.05, and 0.1 μg/ml with 300 mg INH dosing regimen. When patients were characterized with *4/*4, *4/*5, *4/*6, *4/*7, *7/*7, *5/*7, *6/*7, and *6/*6 genotype, differences in PTA among various m/m genotypes were also observed (Figure 5).

## Discussion

In this study, we established for the first time an integrated model for INH and AcINH, using the data derived from healthy Chinese subjects and Chinese tuberculosis patients. We found that various *NAT2* alleles had different impact on INH and AcINH PKs. A Monte Carlo method was used to simulate INH dosing regimen for Chinese patients with different *NAT2* genotypes.

Efficacy and adverse effects of INH and its metabolites in the anti-tuberculosis therapy has been demonstrated. The most important metabolite of INH is AcINH, which is converted to isonicotinic acid (INA) and acetylhydrazine (AcHz) (Boxenbaum and Riegelman, 1976; Lauterburg et al., 1985a). AcHz can be converted either to relatively non-toxic diacetylhydrazine, or a reactive intermediate metabolite through CYP2E1, which may cause toxic hepatitis. INH may also be hydrolyzed to hydrazine (Hz) and INA directly. Oxidation of Hz may also lead to transformation to hepatoxic metabolites; however, this pathway is considered a minor part of the metabolism process, especially in subjects with EM of NAT2 (Boxenbaum and Riegelman, 1976; Lauterburg et al., 1985b). For EM subjects, 7–9%, 39–43%, 0.4–1.6%, and 23% of INH and for PM subjects, 21–32%, 18–27%, 1.1–3.1%, and 3.1–5.1% of INH is excreted as INH, AcINH, AcHz, and diacetylhydrazine, respectively, 24 h after the administration of INH. In contrast, only 0.1–0.4% and 0.5–1.0% of INH is excreted as Hz in EM and PM subjects, respectively (Peretti et al., 1987). INH, AcINH, and its further metabolites account for the major amount of INH recovered from urine. Hence, PKs of INH and AcINH reflected the disposition of INH after administration in a Chinese population sample.

PPK models of INH in different populations have been developed. Both two-compartment (Zvada et al., 2014; Seng et al., 2015; Rodriguez et al., 2019; Jing et al., 2020; Sundell et al., 2020) and one-compartment models have been applied (Cojutti et al., 2017; Aruldhas et al., 2019). The K_a_ was 2.47–6.69 1/h, CL/F of various populations was 5.41–23.7 L/h, and Vd/F was 29.6–72.3 L. Models integrating INH and its metabolite are limited. Seng et al. (2015) established an integrated model for INH, AcINH and INA. A two-compartment model with the best description of the first-order absorption of INH PKs; A two- and one-compartment model was suitable for the description for AcINH and INA data, respectively. They found that NAT2 was a significant covariate for INH clearance, and creatinine clearance was a significant covariate for AcINH clearance. However, only healthy subjects were included in their study. In the present study, for the first time, an integrated model of INH and AcINH was established in both healthy Chinese subjects and tuberculosis patients . Most Chinese subjects could be classified as EM subjects. INH concentration cannot be obtained 12 h after INH administration in these subjects. We found that a one-compartment model was sufficient for most subjects, and the two-compartment model was suggested to be over parameterized. The final integrated model consisted of three compartments, including one deposit compartment and one compartment each for INH and AcINH (Figure 1).

The typical value of CL/F obtained from the integrated model was 18.2 ± 2.45 L⋅h^−1^, which was comparable with previous studies. CL/F of INH included two parts: INH converted to AcINH and direct elimination through urine (Figure 1). F_M_ was used to reflect the part of INH converted to AcINH. It was a key parameter in the integrated model. Since in the present study, INH was administered orally and the part of INH excreted as the parent drug was not determined, F_M_ could not be determined directly. It should be noted that the exact value of clearance (CL_M_/F) and Vd/F of AcINH is difficult to obtain. Using the present integrated model, CL_M_/F could be estimated by multiplying K_30_ with V_3_/F. The CL_AcINH_/F in the present study was estimated as 11.3 L⋅h^−1^, which was similar to that of previous studies.

Polymorphisms in NAT2 activity are responsible for the inter-individual differences in INH PKs (Donald et al., 2007; Seng et al., 2015; Sundell et al., 2020) (14, 29, 30). Most early studies on NAT2 phenotypes classified the subjects as EMs and PMs. Smith et al. found that the activity of NAT2 showed a trimodal distribution (Smith et al., 1997). Kita et al. found that the urinary recovery of INH was lower in healthy volunteers and tuberculosis patients with a higher number of active *NAT2* alleles Kita et al. (2001). Kinzig-Schippers reported that the NAT2 genotypes accounted for 88% variation of INH clearance, which can be presented as: CL/F = 10 + 9 × (number of NAT2*4 alleles) (Kinzig-Schippers et al., 2005). Various *NAT2* SNPs are related to decreased expression and enzyme instability. Hein et al. suggested that catalytic activity of recombinant *NAT2* with 341C/C, 590A/A, and 857A/A genotypes were 10%, 19–42%, and 39–70% of that NAT2 wild type, respectively (Hein et al.,1994; Hein et al., 1995). It can be inferred that although both are classified as PM, the metabolic activity of NAT2 *7/*7 might be 3–6 times higher than that of *5/*5. In our previous study, the influence of various NAT2 SNPs on MR and INH PKs was analyzed using multiple regression. We found that *5(T341→C) allele had a more significant effect than *6(G590→A) and *7(G857→A) on MR_SM2_ and MR_INH_ (Chen et al., 2009). The results showed that various *NAT2* alleles had different impacts on NAT2 activity. In the present study, different *NAT2* genotypes were tested as covariates of CL/F and F_M_. The results showed that OFV decreased, and the model simulation greatly improved. We found significant improvement upon classifying the *NAT2* genotype as wt/wt, m/wt, or m/m, or classification according to various *NAT2* alleles. In the final model, IIV of CL/F and F_M_ was significantly decreased from the basic model (from 56.6% to 28.1%, and 35.3%–9.82%, respectively). CL/F values of *4/*7, *4/*6, *5/*7, and *6/*6 subjects were approximately 74.8%, 54.9%, 34.6%, and 30.1% of *4/*4 subjects. F_M_ was 0.86 in *4/*4 subjects, which was 1.57 fold higher than that of the *4/*6 (0.55) subjects and more than two times higher than that of the *5/*7 (0.36) and *6/*6 (0.35) subjects. It can be deduced that more INH was converted to AcINH in subjects with more active *NAT2* genes. In addition, various NAT2 mutant alleles have different influences on the PKs of INH and AcINH.

A combination of different anti-tuberculosis drugs is usually used for the treatment of tuberculosis; Hence, drug-drug interaction should be considered. According to previous research, rifamycins may induce several metabolic pathways, including CYP3A4, uridine diphosphate glucuronosyltransferases (UGTs), and many transporters. However, there is no evidence of the impact of rifamycin on NAT2 activity and INH PK. On the other hand, pyrazinamide and ethambutol appear to have few significant drug interactions on INH. In our study, 21 healthy subjects were administered a compound preparation including isoniazid, rifampin and pyrazinamide. There was no significant difference in INH PK parameters of these subjects compared with 24 subjects given only INH.

TDM in INH could be helpful in achieving the desired therapeutic target. INH is a concentration-dependent antibiotic; AUC_0–24_ or C_max_ of INH is suggested as the TDM index. Limited sampling strategy models, based on multiple linear regression (MLR) or maximum a posteriori (MAP) analysis, were established for the estimation of AUC in patients who were administered INH. Alshaikheid et al. (2020)established an LSS model, based on 25 patients using the MLR analysis. They found that C_3_, C_2_-C_6_, C_0_- C_1_-C_6_ are suitable for estimation of INH AUC_0–24h_. Cojutti et al. (2017)established a PPK model based on 185 adult tuberculosis patients. LSS models were estimated by the Bayesian method, and C_1_-C_2_-C_5_ and C_1_-C_2_-C_9_ were found to be most suitable. In the present study, concentration-time points were selected based on previous studies using the MAP method. For developed models including C_2_ and C_2_-C_6_, the MPE % were −10.4% and −5.46%, respectively, and MRSE % ranged from 18.5% to 12.4%, indicating that the sampling strategies including C_2_ and C_2_-C_6_ were clinically acceptable. The AUC_0–24_ values were 8.31 ± 4.12, 12.2 ± 5.16, and 27.0 ± 11.4 μg⋅h/ml for patients with wt/wt, m/wt, and m/m genotypes.

AUC_0–24_/MIC is considered as the most important PK/PD index for INH. Dose-scheduling studies demonstrated that antimicrobial capacity of the INH was linked to the AUC_0–24_/MIC (*r* ^2^ = 0.83) (Donald et al., 1997; Jayaram et al., 2004). In the present study, the AUC_0–24_ of INH in Chinese patients with various *NAT2* genotypes was estimated by the Bayesian method. A Monte Carlo method was used to simulate the effect of the standard dose of 300 mg, 600 mg, or 900 mg INH in tuberculosis patients. We found that, for each *NAT2* genotype, the probability of achieving the therapeutic goal was different (Figure 5). Ninety percent of patients with various *NAT2* genotypes achieved the target ratio only when the MIC of INH fell below 0.01 μg/ml. For *Mycobacterium tuberculosis* with an MIC of 0.02 μg/ml, EC90 could be achieved for m/m patients who were administered 300 mg of the drug, m/wt patients who were administered 600 mg of the drug, and wt/wt patients who were administered 900 mg of INH. We also found a difference in PTA among patients with NAT2 m/m genotype. The results showed that different INH dosage regimens could be designed for patients with different *NAT2* genotypes. As there are many more patients with a *NAT2* wt/wt genotypes in the Chinese population, the INH dose should be higher for Chinese subjects. Due to differences in tissue penetration and immunity, INH levels in the site of action, including epithelial cells and macrophages in pulmonary compartments, may exceed those found in plasma. The optimal values for the AUC_0–24_/MIC ratios may be different in the pulmonary compartment and more patients could achieve PTA (Honeybourne et al., 2001). When determining the optimal dose of INH, the efficacy and safety profiles of the drug should also be taken into account in the context of the contributions from other anti-tuberculosis drugs co-administered.

The present study does have a few limitations. First, only INH and AcINH concentration data were obtained. Although AcINH is the most important metabolite, if other metabolites, such as AcHZ and isonicortic acid were included, the established model might have provided more information. Second, there is still high bias of INH or AcINH prediction, especially in the first two hours. Because there were limited samples in the absorption phase, more elaborate absorption models were not applied, causing the PPK model to be inaccurate in predicting K_a_ and C_max_. In addition, factors other than NAT2 genotype were not introduced as covariates in the final model. The influence of those missing factors may partly explain interindividual variation and residual error. Third, the correlation between INH or AcINH and adverse effects may be more valuable. However, we did not obtain that data from patients. Finally, although the determination of the MIC of patients is more valuable for dosing regimen design, we did not do this; hence, only reference data can be used.

## Conclusion

The integrate parent-metabolite population PK model accurately characterized the PKs of INH and AcINH in a Chinese population sample. In a study with a larger number of patients, personalized medicine regimens can be designed, which may be used to increase efficiency and diminish the side effects of INH therapy in Chinese turboculosis patients.

## Data Availability

The original contributions presented in the study are included in the article/Supplementary Materials, further inquiries can be directed to the corresponding author.
